# Correction to Stool‐Test Positivity Proportion and Five‐Year Trend of Intestinal Parasitic Infections Among Symptomatic Students Attending Debre Tabor University Student Clinic, Northwest Ethiopia: A Retrospective Cross‐Sectional Study (2021–2025)

**DOI:** 10.1002/hsr2.73281

**Published:** 2026-09-19

Abeje G, Abebe D, Berhan A, Asefa A, Almaw A, Erkihun M, Yerdaw Y, Tegegne D, Sharew B, Kiros T, Damtie S, Worku S, Malkamu Birhanemaskal, Fentie A, Mengistu WE, Sisay Tadese, Malede B, Mekonnen M, Legesse B, Getie B. Stool‐Test Positivity Proportion and 5‐Year Trend of Intestinal Parasitic Infections Among Symptomatic Students Attending Debre Tabor University Student Clinic, Northwest Ethiopia: A Retrospective Cross‐Sectional Study (2021–2025). Health Sci Rep. 2026;9:e73138. https://doi.org/10.1002/hsr2.73138

The name of the thirteenth author was misspelled and has been updated from Birhanemakel Malkamu to Birhanemaskal Malkamu in the author list and the Author Contributions section.

The online version of this article has been corrected accordingly.

We apologize for this error.

